# Molecular Recognition of Gangliosides and Their Potential for Cancer Immunotherapies

**DOI:** 10.3389/fimmu.2014.00325

**Published:** 2014-07-21

**Authors:** Ute Krengel, Paula A. Bousquet

**Affiliations:** ^1^Department of Chemistry, University of Oslo, Oslo, Norway

**Keywords:** biological membranes, cancer immunotherapy, cell signaling, gangliosides, protein–carbohydrate interactions, glycosphingolipids, sialic acid, tumor-associated antigens

## Abstract

Gangliosides are sialic-acid-containing glycosphingolipids expressed on all vertebrate cells. They are primarily positioned in the plasma membrane with the ceramide part anchored in the membrane and the glycan part exposed on the surface of the cell. These lipids have highly diverse structures, not the least with respect to their carbohydrate chains, with *N*-acetylneuraminic acid (NeuAc) and *N*-glycolylneuraminic acid (NeuGc) being the two most common sialic-acid residues in mammalian cells. Generally, human healthy tissue is deficient in NeuGc, but this molecule is expressed in tumors and in human fetal tissues, and was hence classified as an onco-fetal antigen. Gangliosides perform important functions through carbohydrate-specific interactions with proteins, for example, as receptors in cell–cell recognition, which can be exploited by viruses and other pathogens, and also by regulating signaling proteins, such as the epidermal growth factor receptor (EGFR) and the vascular endothelial growth factor receptor (VEGFR), through lateral interaction in the membrane. Through both mechanisms, tumor-associated gangliosides may affect malignant progression, which makes them attractive targets for cancer immunotherapies. In this review, we describe how proteins recognize gangliosides, focusing on the molecular recognition of gangliosides associated with cancer immunotherapy, and discuss the importance of these molecules in cancer research.

## Introduction

**Figure 1:**
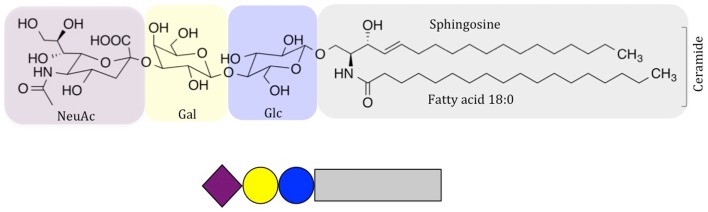
**Schematic drawing of NeuAc GM3, a common ganglioside in vertebrate tissues**. Carbohydrate symbols follow the nomenclature of the Consortium for Functional Glycomics (2); purple diamond – *N*-acetylneuraminic acid; yellow circle – D-galactose; blue circle – D-glucose.

Few lipid species included in biological membranes have received as much attention as glycosphingolipids (GSLs), and especially gangliosides, sialic-acid-containing GSLs. They were discovered by Ernst Klenk in the 1940s, who proposed the term “ganglioside” due to the abundance of these molecules in “Ganglionzellen” (neurons). Gangliosides were later classified by Svennerholm according to the number of sialic-acid residues and chromatographic mobility (1). In contrast to glycerolipids, the lipid anchor in sphingolipids builds on the long-chain amino alcohol sphingosine, which is coupled *via* its amino group to a fatty acid to form ceramide (Figure 1). In gangliosides, the ceramide anchor is linked to a hydrophilic glycan head group, which is characterized by the presence of one or more sialic-acid residues (carbohydrates with a nine-carbon backbone and a carboxylic acid group); however, there is large variability of this structure. One example, the GM3 ganglioside, abundant in almost all healthy tissues, is shown in Figure 1. The large structural variability is related to developmental stage and cell type, and hundreds of gangliosides are known today (3–5). Variations in carbohydrate structure alone account for over a 100 different structures, and this number significantly increases, when ceramide variations are taken into account (4–7). Accumulating evidence indicates that many cellular events, including differentiation, growth, signaling, interactions, and immune reactions are highly influenced by gangliosides, and that these molecules may also cause malignancies. Positioned in the plasma membrane, gangliosides interact with other lipids and proteins, both laterally in the membrane and *via* their head groups, acting as cellular receptors that can be recognized by antibodies and other ganglioside-binding molecules. Here, we highlight the function and molecular interactions of gangliosides with high clinical significance.

## Gangliosides – General Architecture, Cellular Localization, and Biosynthesis

Gangliosides consist of a lipid anchor, the ceramide, decorated by a glycan head group of various complexity. In cells, gangliosides are mainly found in the outer leaflets of the plasma membrane. Together with sphingomyelin and cholesterol, they form membrane microdomains, which play important roles in cell–cell communication and signal transduction (8–10). The synthesis of gangliosides starts in the ER compartment with the synthesis of the ceramide, the common precursor of all GSLs. Aided by the ceramide-transfer protein, CERT, ceramide is then transferred to the Golgi apparatus, and thereafter converted to glucosylceramide (GlcCer) (11). Subsequently, other carbohydrate residues are attached, one by one, catalyzed by glycosyltransferases, as described below (12, 13). The glycosyltransferases are specific to the sugar residues that they transfer and are grouped into families according to their specificity. Interestingly, all glycosyltransferase promoters lack the TATA sequence, and hence do not have any core promoter element characteristic for house-keeping genes. Although some indications relate their transcription to complex developmental and tissue-specific regulation, very little is known about how glycosyltransferases are regulated (14). The molecular products are further subject to remodeling, by sialidases, sialyltransferases, and other enzymes, followed by vesicle sorting and fusion with the plasma membrane (15). Gangliosides are assumed to recycle to the plasma membrane from early endosomes, and a degradation process is thought to take place at the late endosomal level (16).

**Figure 2:**
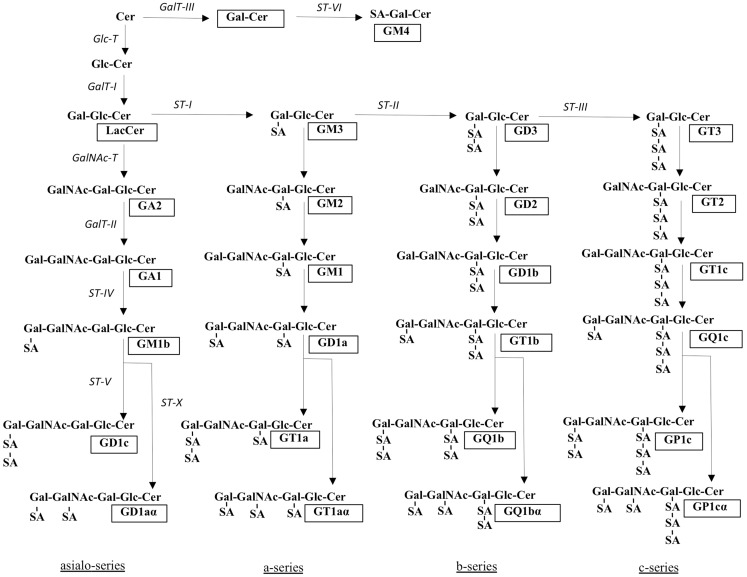
**Structures and biosynthetic pathways of gangliosides**. The glycosyltransferases catalyzing the synthesis of gangliosides are shown in italics. Cer, ceramide; SA, sialic acid. Ganglioside nomenclature [according to Svennerholm (1)] is shown in boxes. Adapted from Ref. (5).

The biosynthetic pathways of gangliosides are shown in Figure 2. After formation of the initial glucosylceramide, a galactose moiety is added to GlcCer to yield lactosylceramide (LacCer), the common precursor for almost all gangliosides (except GM4). Addition of one sialic-acid residue to LacCer subsequently converts this precursor molecule to GM3. This reaction is catalyzed by sialyltransferase I (ST-I) or GM3 synthase. In the same manner, GD3 and GT3 can be generated by further addition of sialic-acid residues, catalyzed by ST-II or GD3 synthase and ST-III or GT3 synthase, respectively. The number of sialic-acid residues linked to the inner galactose residue (0, 1, 2, or 3) classify the gangliosides into asialo, a-, b-, or c-series (Figure 2), however, only trace amounts of gangliosides from the asialo- and c-series are found in adult human tissue (17).

## Gangliosides – Biological Function and Exploitation by Pathogens

Gangliosides are key molecules in cellular recognition and signaling. They are primarily present in the plasma membranes of vertebrates, but have recently also been found in nuclear membranes, recognized as functionally important constituents (18, 19). Knock-out studies in mice have been essential for revealing the functions of gangliosides, especially in embryonic development and differentiation. For example, Yamashita et al. observed that mouse embryos carrying a knock-out in the glycosylceramide synthase enzyme did not survive more than 7.5 days (20). Other examples are studies of mice with a knock-down of GM3 synthase and GM2/GD2 synthase, which exhibit increased insulin sensitivity and decreased ability to repair nervous tissues, respectively (21, 22).

Because of the tight packing of lipids in membranes, gangliosides associate with other types of lipids, forming membrane subcompartments such as lipid rafts, to which specific proteins can associate (8, 23, 24). The organization of gangliosides in membranes will be further discussed in the Section “Organization and Presentation of Gangliosides in Biological Membranes.” Since gangliosides have the ability to interact with both sugars and proteins (see Sections “Gangliosides – Structure and Molecular Recognition”, “Organization and Presentation of Gangliosides in Biological Membranes”, and “Effect of Gangliosides on Membrane Proteins and Cellular Signaling”), a large range of events can be triggered or inhibited by these molecules. Cell growth, migration, differentiation, adhesion, and apoptosis are some examples (25, 26). The terminal sialic-acid residue(s) in particular are targets for many important intercellular interactions, but can also be exploited by pathogens that use these residues as a docking station to enter the cell (27).

Various pathogens, from viruses to bacteria and parasites, recognize sialic-acid residues on host cell membranes, several of these known to cause cancer. The most common recognition module is NeuAc; in addition, NeuGc and 9-*O*-acetylated sialic acids are also well-known receptors (28, 29). Examples of viral pathogens recognizing gangliosides are the influenza virus (30), simian virus 40 (SV40) (31), and polyomavirus (32, 33). Bacteria interact with gangliosides *via* toxins and adhesins, with the cholera toxin (34) and the Sialic-acid binding adhesin from the Class 1 carcinogen *Helicobacter pylori*, SabA (35, 36), being prominent examples. Gangliosides may also suppress natural killer (NK) cell cytotoxicity, through interaction with Siglec-7 (sialic-acid binding immunoglobulin-like lectin 7), as elaborated further in the Section “Gangliosides and Cancer.”

## Gangliosides – Structure and Molecular Recognition

**Figure 3:**
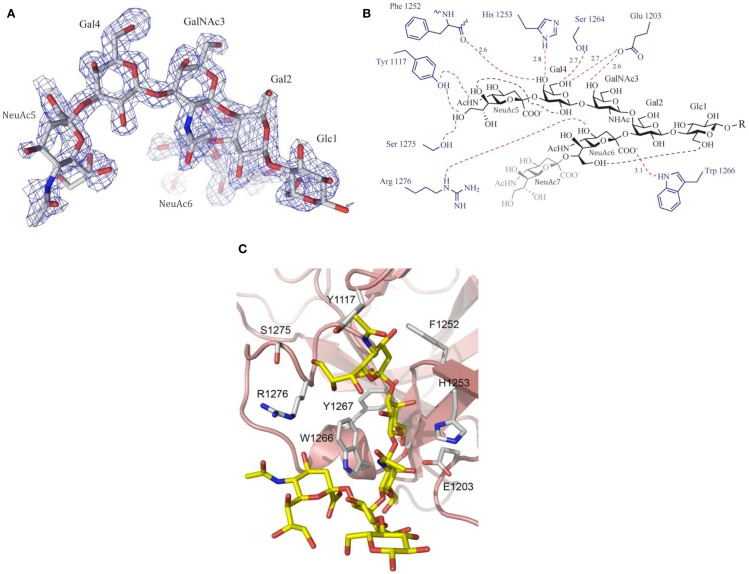
**Example of ganglioside recognition [here: GT1b (analog) and its interaction with botulinum neurotoxin type A (BoNT/A)]**. **(A)** Experimental electron density (Fo–Fc omit map) of the ganglioside head group. **(B)** Schematic drawing of the interactions between GT1b and BoNT/A. Hydrogen bonds are shown as dotted lines (red: intermolecular interactions; black: intramolecular carbohydrate–carbohydrate interactions). **(C)** Close-up view of the ligand-binding site. Please note the aromatic stacking interactions with Trp 1266 and Tyr 1117. Printed with permission from Ref. (40).

The molecular recognition of carbohydrates, with their large number of hydroxyl groups, is dominated by hydrogen bonds, with the binding specificity determined by the recognition of the characteristic OH-scaffolds of different sugars (37, 38). Many of these interactions are water-mediated, and sometimes, metal ions are involved. In addition, hydrophobic interactions contribute significantly to carbohydrate recognition, which may involve methyl groups such as in the monosaccharide fucose or the stacking against exposed hydrophobic patches of the sugar rings. A particularly typical molecular recognition mechanism of carbohydrates involves the CH-π stacking of sugar rings against the side chains of aromatic amino acids (so-called “aromatic stacking interactions”), promoted by weak hydrogen bonds (39) (Figure 3).

Gangliosides are characterized by the presence of at least one sialic-acid residue, which in contrast to many other sugars is charged. This charge can be exploited by salt bridges with positively charged residues, but this is not necessarily the case (and in fact quite rare). The carboxylate group is often not even the most important recognition motif. For example, the fingerprint of the most common sialic acid, *N*-acetylneuraminic acid (NeuAc), which is derived from pyruvate and *N*-acetylmannosamine, generally involves the recognition of the *N*-acetyl group and the adjacent 4-OH-group, originating from mannose (which corresponds to 3-OH in hexoses) (41). Further H-bonding interactions are provided by the sialic-acid glycerol chain (also originating from mannose), which is recognized by a conserved binding motif common to a number of viral and bacterial sialic-acid binding proteins (42). In addition, conformer selection and clustering play important roles for the molecular recognition of gangliosides, as shown for example for the recognition of GM1 by the cholera toxin or galectin-1 (34, 43–45).

Carbohydrates in general are flexible molecules, but due to internal carbohydrate–carbohydrate interactions, the influence of the lipid anchor, or due to interactions with other molecules in the immediate neighborhood, rigid molecular epitopes may arise. As gangliosides are localized in the plasma membrane, the presentation of the carbohydrate epitopes in particular depends on the interaction with other lipids (8). However, the structural characterization of anchored gangliosides is difficult to achieve. State-of-the-art lipid simulations are described by Vattulainen and Róg (46), but these often fail to take the glycan head groups into account. Nevertheless a few studies have been undertaken that do just that. One interesting example is the atomic-resolution conformational analysis of GM3 in a bilayer composed of dimyristoylphosphatidylcholine (DMPC) (47). Two known GM3-binding proteins [sialoadhesin, PDB ID: 1QFO (48), and wheat germ agglutinin, PDB ID: 2CWG (49)] were studied in order to evaluate the importance of carbohydrate accessibility and ganglioside recognition. Probing the presentation and dynamics of the glycan head group, DeMarco and Woods observed significantly altered accessibility of the less exposed carbohydrate residues Gal and Glc, even though the internal structural properties for membrane-bound versus soluble GM3 were unchanged. On the other hand, the terminal NeuAc-residue remained almost fully exposed. The difference in accessibility is likely of considerable importance for the initial recognition of GM3 by a receptor protein, although subsequent recognition events may include the glycan residues embedded deeper in the membrane. The less exposed residues may also indirectly affect recognition, by ceramide–Glc and Glc–Gal rotations, altering NeuAc presentation. Furthermore, the hydrophobic ceramide together with the polar Glc residue may regulate the insertion depth.

## Organization and Presentation of Gangliosides in Biological Membranes

Cellular membranes serve both as segregation barriers and as facilitators of cellular communication. Positioned in the cell membrane, lipids interact laterally with other membrane components (lipids or membrane proteins), and also serve as cellular receptors, through their exposed head groups. In the past decade many studies have focused on the lateral characterization of membranes and it is now well-established that highly unsaturated components, like glycerophospholipids, provide the membrane with flexibility, while saturated components, such as GSLs, create order in biological membranes (10). Furthermore, the shape and length of the lipids determine the shape, size, and stability of cellular membranes (50). The ceramide part of gangliosides is characterized by a rigid and planar structure, composed of saturated acyl chains, which can be more tightly packed. Together with other membrane sphingolipids and cholesterol, they can segregate and form dynamic nanoscale “clusters”, also called lipid rafts (8, 24, 51), to which specific proteins associate, hitching a ride.

Apparently, the density of GSLs can also influence their structure, affecting antigen specificity. For example, an antibody established by immunizing mice with syngeneric B16 melanoma, named M2590, reacted only with melanoma and not with healthy tissues (52). Remarkably, the target epitope was later identified as GM3, an abundant ganglioside in membranes of normal cells (53). Further studies showed that a ganglioside density above a threshold value was required for reactivity, suggesting that this antibody recognized more densely packed GM3 (54). These results indicate that ganglioside antigens can be differently organized in tumor cells compared to normal cells and that some ganglioside antigens are fully antigenic when organized in clusters, but fail to bind antibodies when their density is under a threshold value (54, 55).

**Figure 4:**
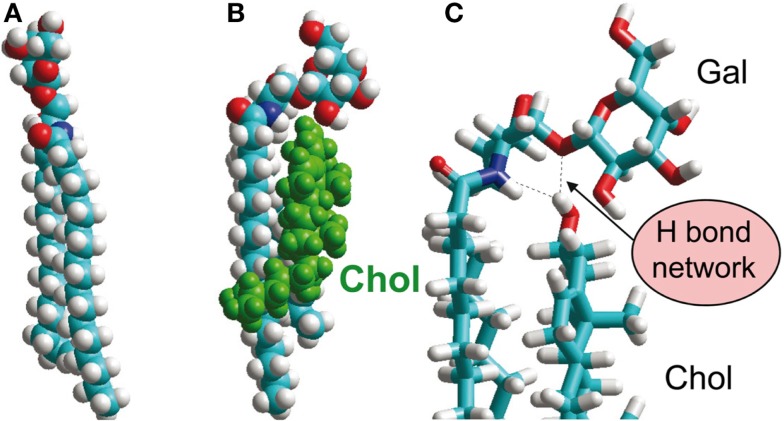
**Glycosphingolipid interaction with cholesterol, an important constituent of lipid rafts**. **(A)** GalCer, extended conformation. **(B,C)** GalCer, tilted conformation, induced by H-bonding interactions with cholesterol OH-group, shown in (**C)** [**(A,B)**: space-filling representation, **(C)**: stick representation]. Printed with permission from Ref. (56), in an extension of earlier work by Nyholm et al. (58).

How can this be explained? This brings us back to the structural characterization of GSLs in biological membranes. One example has already been described [GM3 in DMPC bilayer; (47)]. Two other interesting studies evaluate the effect of cholesterol on GSL structure (56, 57), building on earlier work by Pascher and coworkers (58). Notably, cholesterol was found to introduce a tilt in the glycolipid head group from a conformation almost perpendicular to the membrane surface to an alignment parallel to the membrane (Figure 4). The culprit appears to be an H-bonding network involving the cholesterol OH-group, the sphingosine amide, and the oxygen of the glycosidic bond (56). Similar lipid-raft-specific conformational changes of GSLs may be critical for the entry of bacterial toxins or viruses into host cells (8, 59).

Glycosphingolipids are not always fully accessible, however. Their short head groups may be hidden in the “jungle” of membrane proteins or even masked by sialic-acid binding proteins positioned near the GSLs in the membranes (i.e., in *cis*). Such a scenario is postulated, e.g., for Siglecs, a family of lectins that modulate innate and adaptive immune functions. *Trans* interactions may still occur, e.g., for higher-affinity ligands that can out-compete the *cis* ligands, however, in general, accessibility will be reduced.

## Effect of Gangliosides on Membrane Proteins and Cellular Signaling

It has been suggested that also the activation of membrane proteins can be influenced by lipid cluster association. In addition to lateral interaction with the lipid tails in the cell membrane, such interactions may exploit the unique properties of sphingolipids, bearing a carbonyl oxygen, a hydroxyl group, and an amide nitrogen, thus being able to act as both H-bond donors and acceptors (60). As described in the previous section, gangliosides and other GSLs may further cause conformational changes of the glycan head group, which may either interact directly with amino acids of the extracellular part of the protein or alternatively interact with the sugar residues of a glycosylated protein, affecting protein activity.

Most growth factor receptors are known to be regulated by gangliosides (9). Here, we will discuss two examples of membrane proteins important for cancer research and immunotherapy: the epidermal growth factor receptor (EGFR) and the vascular endothelial growth factor receptor (VEGFR) (Table 1). A number of cancers are characterized by hyper-activated EGFRs, either caused by mutations or over-expression (61–63). Another important factor for tumor progression is the growth of new blood vessels. Tumor cells produce and release the growth factor VEGF, stimulating the VEGFR, and ultimately resulting in proliferation and migration of vascular endothelial cells (64).

**Table 1:** **Gangliosides affecting the growth factor receptors EGFR and VEGFR**.

Ganglioside	Growth factor receptor	Reference
GM3	EGFR	(65–68)
GM1	EGFR	(68, 69)
GM2	EGFR	(70, 71)
GM4	EGFR	(70)
GD3	EGFR	(70, 72)
GD1a	EGFR	(68, 73)
GT1b	EGFR	(68)
GM3	VEGFR	(74, 75)
GD1a	VEGFR	(75, 76)
GD3	VEGFR	(77)

The EGFR is known to undergo ligand-dependent dimerization, resulting in an autophosphorylation of tyrosine residues at the C-terminal tail of the protein (78). This initiates downstream signaling, leading to adhesion, cell migration, and proliferation (79). More recently, the EGFR has also been shown to undergo ligand-independent dimerization, a phenomenon that is poorly understood (80). Such ligand-free dimers can also be functionally active, but this is not always the case.

Several membrane ligands have been shown to affect signaling by the EGFR and the VEGFR. The GM3 ganglioside, a well-known regulator of the insulin receptor (81), has an inhibitory effect on both the EGFR and the VEGFR, while the ganglioside GD1a strongly induces VEGFR-2 activation (26, 66, 70, 75, 82, 83). Moreover, the proangiogenic effects of GD1a can be efficiently reduced by GM3 (75). GM3 has been suggested to inhibit VEGFR-2 activation by blocking both growth factor binding and receptor dimerization through direct interaction with the extracellular domain of the VEGFR (74). The molecular interaction between the EGFR and GM3 is not fully elucidated, although it has been studied extensively. It has been shown that the inhibition of EGFR activation by GM3 involves the binding of the ganglioside to the GlcNAc-terminated *N*-glycans on the EGFR, suggesting carbohydrate–carbohydrate interactions (65, 67, 84, 85). In addition, increasing evidence points to the integral importance of ganglioside organization in the membrane for signal transduction (affecting the localization and activation of growth factor receptors). For example, recent computer simulations of the EGFR embedded in the membrane suggest that membrane lipids, especially anionic species, interact extensively with the EGFR (86). These interactions are more pronounced for the inactive EGFR, due to electrostatic interactions with the EGFR’s intracellular domain, which may explain the inhibitory effect of GM3 on EGFR activation.

Cellular biological membranes are complex and the dynamics difficult to study. Even small modifications like the fluorescent labeling of lipids may critically affect bulk membrane properties as well as ligand–receptor interactions in biological environments (87). To generate a more controllable system, Coskun et al. reconstituted EGFR into proteoliposomes with defined lipid composition, with either uniform liquid-disordered (ld) membrane phases or a combination of disordered and ordered (ld/lo) domains. Adding gangliosides to this system, they found that GM3 had a strong inhibitory effect on EGFR activation, without interfering with ligand-binding, but in ld/lo proteoliposomes only (66). It would be of significant clinical interest to investigate how targeting GM3 by immunotherapy affects EGFR and VEGFR signaling, and whether the presence of both targets (GM3 clusters and EGFR/VEGFR) affect antibody efficiency and affinity.

## Gangliosides and Cancer

Gangliosides play important roles in many normal physiological processes, such as cell growth, differentiation, and embryogenesis (20), but also in pathological events like cellular malignancy and metastasis (88) (see Table 2 for examples of gangliosides expressed in human cancer cells). Tumor formation results from autonomous uncontrolled proliferation of neoplastic cells, while metastasis occurs when tumor cells are released from the primary tumor and continue to proliferate at a distant site. Multiple factors affect these processes, in which gangliosides may serve both as inhibitory and stimulating molecules. For example, it has been shown that highly metastatic melanoma cells have high expression levels of GD3. This is in contrast to poorly metastatic cells or the normal counterpart, melanocytes, which express very low levels of GD3 (89–91), suggesting a role of GD3 in transforming melanocytes into melanomas and promotion of metastasis. Gangliosides may suppress NK cell cytotoxicity through interaction with Siglec-7, which preferentially binds to gangliosides of the b-series, as found for cells engineered to overexpress GD3 (92). The high expression levels of the GD3 ganglioside in melanoma may hence reflect the suppressed efficiency of NK cell cytotoxicity against these tumor cells. The function of gangliosides as suppressors of the anti-tumor immune response is well-documented in many studies, with tumor-associated gangliosides reported to down-regulate the activity of T and B cells, NK cytotoxicity and active dendritic cells, among others (93–95). For instance, T-cell dysfunction is promoted by the GM2 ganglioside, however, an antibody targeting GM2 was able to block 50–60% of T-cell apoptosis (94).

**Table 2:** **Gangliosides expressed in human cancer cells**.

Ganglioside	Structure	Cancer type	Reference
NeuAc GM3	αNeu5Ac(2-3)βDGal(1-4)βDGlc(1-1)Cer	Melanoma, NSCLC, breast carcinoma, renal carcinoma	(89, 96–100)
NeuGc GM3	αNeu5Gc(2-3)βDGal(1-4)βDGlc(1-1)Cer	Colon cancer, retinoblastoma, melanoma, breast carcinoma, neuroectodermal cancer, Wilms tumor	(98, 99, 101–104)
GM2	βDGalNAc(1-4)[αNeu5Ac(2-3)]βDGal(1-4)βDGlc(1-1)Cer	Melanoma, neuroblastoma, SCLC, t-ALL, breast carcinoma, renal carcinoma	(74, 96, 99, 100, 105–107)
GM1	βDGal(1-3)βDGalNAc[αNeu5Ac(2-3)]βDGal(1-4)βDGlc(1-1)Cer	SCLC, renal carcinoma	(99, 106)
GD3	αNeu5Ac(2-8)αNeu5Ac(2-3)βDGal(1-4)βDGlc(1-1)Cer	Melanoma, neuroblastoma, glioma, SCLC, t-ALL, breast carcinoma	(25, 89, 96, 97, 105, 107–111)
GD2	βDGalNAc(1-4)[αNeu5Ac(2-8)αNeu5Ac(2-3)]βDGal(1-4)βDGlc(1-1)Cer	Melanoma, neuroblastoma, glioma, SCLC, t-ALL	(89, 96, 97, 105–109)

*αNeuAc = 5-acetyl-α-neuraminic acid, αNeuGc = 5-glycolyl-α-neuraminic acid, βDGal = β-d-galactopyranose, βDGalNAc = *N*-acetyl-β-d-galactopyranose, βDGlc = β-d-glucopyranose, Cer = ceramide, NSCLC = non-small-cell lung carcinoma, SCLC = small-cell lung carcinoma*.

Gangliosides are also shed from the tumor to the microenvironment in greater quantities than normal cells. Shed gangliosides can interact with proteins or be incorporated into the membrane of other cells, leading to signaling events or interactions with healthy cells (112–114). For example, the addition of exogenous GD3 to the culture medium of glioma cells was found to stimulate the release of VEGF (115). Taken together, these observations suggest a multitude of mechanisms by which tumor-associated gangliosides may contribute to malignancy and cancer progression.

Many of the tumor-associated gangliosides are also found in normal healthy tissues, but are over-expressed in tumors, while other antigens are only found in cancer cells. An interesting example is the sialic-acid NeuGc, which is found in several tumor types, such as melanoma and breast cancer (116). Among all variants of sialic acids, NeuAc and NeuGc are the most abundant; however, humans are a notable exception. Due to a 92-bp deletion in the gene coding for CMP-NeuAc hydroxylase (*cmah*), humans lack a functional enzyme required for generation of NeuGc (117, 118). Nevertheless, NeuGc is present in fetal tissues and malignant cells (99, 119, 120). For this reason, NeuGc was assumed to classify as an “onco-fetal” antigen, being expressed in the fetus, suppressed during adult life and re-expressed in malignant cells. However, since humans lack the putative active site of the enzyme, other explanations must lie at the heart of this change in carbohydrate profile. Diet incorporation, hypoxic conditions, and endogenous metabolic mechanisms are currently being discussed as possible origins of the increased levels of NeuGc (116, 121–124). Getting to grips with the high NeuGc-ganglioside levels is important, since this property appears to correlate with a poor prognosis. Specifically, recent studies indicate that non-small-cell lung cancer (NSCLC) patients with high NeuGc-ganglioside expression exhibit a low overall survival rate and a significantly lower progression-free survival rate (125). These findings are consistent with recent experiments demonstrating that the silencing of the *cmah* gene in NeuGc GM3-expressing L1210 mouse lymphocytic leukemia B cells caused a shift to NeuAc GM3 expression and a concomitant reduction of tumorigenicity (126).

Interestingly, it has been shown that serum from healthy humans contains antibodies recognizing glycoconjugates exhibiting NeuGc (127, 128). These antibodies are called Hanganutziu–Deicher (HD) antibodies, and were first described by Hanganutziu (129) and Deicher (130) [as cited in Ref. (131)] independently in the 1920s. HD antibodies attract complement molecules to malignant cells (132, 133). The level decreases with age, which may correlate with an increased cancer risk at higher age (133). Characteristic for natural antibodies is that they recognize highly conserved antigens (134). Importantly, auto-antibodies against tumor-associated antigens can arise and be detected early, before symptoms occur, and hence have potential for early diagnosis (135–137). In line with this hypothesis, a recent study reported that healthy donors exhibited low levels of anti-NeuGc GM3 antibodies (decreasing with age), while these antibodies were absent in NSCLC patients (138).

## Ganglioside-Based Therapy

Cancer immunotherapy is a highly promising approach to cancer treatment, which has been gaining grounds only recently (139). In contrast to traditional therapies like chemo- or radiation-therapy, immunotherapies constitute a much more targeted approach that promises higher specificity while eliciting fewer side effects. As the name states, this type of therapy uses the immune system to treat cancer. There are two main approaches (139, 140): (i) tumor-associated antigens or derivatives or mimics of these may be used as active therapeutic vaccines, priming the body to launch an immune attack against these molecules and hence the tumor cells (overcoming the body’s tolerance of self-antigens); (ii) alternatively, antibodies may be used for passive immunotherapy, either coupled to toxins, radioactivity or on their own, relying on processes like antibody-dependent cell-mediated cytotoxicity (ADCC) or complement-dependent cytotoxicity (CDC). In both cases, effective immunotherapy relies on the choice of the antigen. Notably, in a recent project for prioritization of cancer antigens, 4 of the 75 selected antigens were gangliosides (GD2, GD3, fucosyl-GM1, and *N*-acetyl GM3), and additional targets, like the EGFR and the VEGFR, are known to interact with gangliosides (141).

Several antibodies targeting tumor-associated gangliosides are currently under investigation in pre-clinical or clinical studies, also including molecular vaccines. One example, the antibody 3F8, targets GD2, which is highly expressed in aggressive cancer, such as pediatric neuroblastoma (142). Other examples are 14F7 and chP3, both of which specifically recognize NeuGc GM3, discriminating it from the highly similar NeuAc GM3. So far, no crystal structures of these complexes have been reported, however, computer docking studies, *in silico* site mapping and phage display studies are contributing to reveal the recognition mechanisms of these promising tools (143–146). In addition, two NeuGc-ganglioside-based vaccines are currently tested in clinical trials (phase III); these are Racotumomab, an anti-idiotypic antibody^1^ registered and launched in Cuba and Argentina under the trade name Vaxira (147) and NeuGc GM3/VSSP, a NeuGc GM3 ganglioside conjugated into very small proteoliposomes. In the ongoing clinical trials, the NeuGc GM3/VSSP and Racotumomab vaccines show efficacy and are well-tolerated by patients with advanced cutaneous melanoma (148) and NSCLC (149), respectively. This represents a significant step forward from the first, unsuccessful, attempt of developing a ganglioside-based vaccine – the GMK (GM2-based) vaccine for melanoma (150, 151). These molecules are part of a growing arsenal of targeted molecular weapons against cancer, which may be used as stand-alone therapy, but will more likely be employed as adjuvant therapy, in combination with or following standard treatment such as surgery, radiation, or chemotherapy. For example, based on the important roles of NeuGc GM3 and the EGFR for tumor cell immune evasion and proliferation, a combination therapy targeting both molecules may provide a rationale for fighting tumor cells. This combination is currently tested using Racotumomab and a vaccine targeting EGF in NSCLC patients, showing, so far, promising clinical results (152).

## Conclusion

Today, we are still far from fully understanding the roles, structures, and mechanisms of gangliosides in biological systems, and only at the beginning of the exploitation of these molecules in potential therapies. However, the importance of these molecules is evident, and technology development is picking up pace (7, 46, 153, 154). We are looking forward to a bright future, in which gangliosides are fully appreciated, and unfold their full potential in targeted therapies.

## Conflict of Interest Statement

The authors declare that the research was conducted in the absence of any commercial or financial relationships that could be construed as a potential conflict of interest.

## Footnotes

^1^*Explanation*: Antibodies (Ab2) raised against the primary antibody (Ab1, generated in the original immune response against cancer antigens) are also called “anti-idiotypic” antibodies. They may have potential as therapeutic vaccines if they give rise to a third class of antibodies (Ab3) that resembles the primary antibody (Ab1).
